# Body composition and immunonutritional status in patients treated with pressurized intraperitoneal aerosol chemotherapy (PIPAC) for gastrointestinal peritoneal metastases: a prospective single-center analysis

**DOI:** 10.1515/pp-2021-0142

**Published:** 2022-03-01

**Authors:** Stefano Rotolo, Andrea Di Giorgio, Marco Cintoni, Emanuele Rinninella, Marta Palombaro, Gabriele Pulcini, Carlo Alberto Schena, Vito Chiantera, Giuseppe Vizzielli, Antonio Gasbarrini, Fabio Pacelli, Maria Cristina Mele

**Affiliations:** Dipartimento di Scienze Mediche e Chirurgiche, UOC Chirurgia del Peritoneo e del Retroperitoneo, Fondazione Policlinico Universitario A. Gemelli IRCCS, Rome, Italy; Dipartimento di Discipline Chirurgiche, Oncologiche e Stomatologiche (Di.Chir.On.S.), Università degli Studi di Palermo, Palermo, Italy; Dipartimento di Scienze Mediche e Chirurgiche, UOC di Nutrizione Clinica, Fondazione Policlinico Universitario A. Gemelli IRCCS, Rome, Italy; Dipartimento di Scienze Mediche e Chirurgiche, UOSD di Nutrizione Avanzata in Oncologia, Fondazione Policlinico Universitario A. Gemelli IRCCS, Rome, Italy; Dipartimento per la salute della Donna e del Bambino e della Salute Pubblica, UOC Ginecologia Oncologica, Fondazione Policlinico Universitario A. Gemelli IRCCS, Rome, Italy; Dipartimento di Scienze Mediche e Chirurgiche, UOC Medicina Interna e Gastroenterologia, Fondazione Policlinico Universitario A. Gemelli IRCCS, Rome, Italy; Dipartimento di Medicina e Chirurgia Traslazionale, Università Cattolica Del Sacro Cuore, Rome, Italy

**Keywords:** body composition, clinical nutrition, pressurized intraperitoneal chemotherapy, prognostic nutritional index, skeletal muscle index

## Abstract

**Objectives:**

Pressurized intraperitoneal aerosol chemotherapy (PIPAC) is a novel drug administration method with promising efficacy for the treatment of peritoneal metastases (PM). This study aimed to evaluate the prognostic value of an immunonutritional assessment on the feasibility, safety, and survival in this setting.

**Methods:**

Data of PM patients undergoing PIPAC between September 2018 and May 2020 were prospectively recorded. A CT scan-derived body composition assessment was performed for each patient.

**Results:**

Fifty-one patients were enrolled, of which 30 (58%) underwent multiple PIPAC cycles, with a pathological response rate of 55%. Prognostic nutritional index (PNI) and neutrophil-to-lymphocytes predicted completion of more than one PIPAC cycle, with a cut off of 36.5 and 4.8 respectively. Muscle attenuation and body fat tissues were associated with pathological response. At multivariate Cox regression analysis, only the presence of a low PNI (HR 2.41, 95% CI 1.08–5.46) was significantly associated with a worse OS.

**Conclusions:**

A pretreatment immunonutritional assessment may provide valuable information for PIPAC patients’ selection and survival, while body composition parameters are able to predict pathological response. Further larger studies are needed to validate the role of these biomarkers in tailoring the treatment and monitoring PM patients undergoing PIPAC.

## Introduction

Pressurized intraperitoneal aerosol chemotherapy (PIPAC) is a novel locoregional chemotherapy recently proposed for patients affected by peritoneal dissemination from gastrointestinal and gynecological cancers. Several phase-I and phase-II studies reported reassuring safety data and high antitumoral efficacy of PIPAC alone or in combination with systemic chemotherapy [1], [2], [3]. Based on laparoscopy, PIPAC may be repeated several times, enhancing the chance to hit active neoplastic cells. Despite defined PIPAC schedules being lacking, it has been proposed that multiple administrations should be carried out to exert the best antiblastic efficacy [4], [5], [6]. Unfortunately, the reported rate of completing a three-cycle course hardly reaches 50% in published cohorts. This may not be surprising, given that PIPAC has been mostly administered in late-stage diseases in a palliative setting [7], [8], [9], [10], [11]. The selection of patients undergoing PIPAC relies on several factors and it is decided on a case-by-case basis in most studies, as precise PIPAC indications still need to be accurately defined [12].

A large body of evidence disclosed the role of the immunonutritional status in oncological patients, which entails their capacity to cope with surgical and antiblastic treatments. Several scores based on blood test, including neutrophil-to-lymphocyte ratio (NLR), platelet-to-lymphocyte ratio (PLR), and prognostic nutritional index (PNI), have been reported to correlate with postoperative complications and survival in patients with gastrointestinal cancer [13]. NLR and PLR, as markers of systemic inflammatory response, are closely associated with cancer development, progression, and metastasis and have been used as prognostic indicators in many solid tumors [14], [15], [16]. Moreover, in the oncological setting, the computed tomography (CT) scan-derived body composition analysis has grown in interest in the last decade, due both to the high availability of CT scans in neoplastic patients [17, 18], and to the association of CT-derived parameters with an increased risk of chemotherapy toxicity and poor survival [19].

Nutritional status is a major determinant of surgical and oncological outcomes. In 2016, the Global Leadership Initiative on Malnutrition (GLIM), through the collaboration of the leading nutrition societies, defined a standardised approach for the diagnosis of malnutrition. The evaluation starts with the analysis of the “risk” status using any of the already validated screening tools. The second step is the assessment of malnutrition and its severity. To do so, different criteria have been identified and classified into phenotypic and etiological. Phenotypic criteria include involuntary weight loss, reduced BMI and/or reduced FFM, measured through validated procedures, such as bioelectrical impedance analysis and fat free mass index (FFMI). Etiological criteria are reduced food intake or assimilation and inflammation or disease burden. To diagnose malnutrition at least one phenotypic criterion and one etiologic criterion should be present. Once diagnosed, malnutrition is stratified into moderate or severe depending on phenotypic criteria.

This study aimed to evaluate the value of pretreatment immunonutritional status and CT-derived body composition parameters on feasibility, safety, efficacy, and survival of patients undergoing PIPAC for gastrointestinal peritoneal metastases (PM).

## Materials and methods

### Study design

We prospectively recorded the clinical data of patients undergoing PIPAC for PM of gastrointestinal origin between September 2018 to May 2020 at the Foundation Policlinico A. Gemelli IRCCS, Rome, Italy. For each patient age, gender, American Society of Anesthesiology (ASA) score, Eastern Cooperative Oncology Group Performance Status (ECOG PS), previous oncological and surgical history, were prospectively recorded. Laboratory test results included absolute counts of white blood cells, absolute neutrophil count (NEU), absolute lymphocyte count (LYM), platelet count (PLT), and albumin (ALB) levels. Moreover, starting from laboratory data taken, the following parameters were calculated: PNI [20]: ALB [g/L] + 0.005 × LYM, NLR: NEU/LYM, and PLR: PLT/LYM. At baseline, a complete nutritional evaluation, including weight, height, body mass index (BMI), was carried out. All patients who had already received any type of nutritional intervention (i.e. oral nutritional supplements, enteral nutrition, parenteral nutrition, etc.) were excluded from the analysis. CT scan images, taken within one month before the PIPAC procedure, were collected for body composition assessment. The following perioperative data were recorded: the number of PIPAC cycles administered per patient and the incidence of no-entry at laparoscopy, peritoneal cancer index (PCI), ascites volume, intraoperative complications, length of hospital stay, readmission rate, 30-days postoperative adverse events according to the National Cancer Institute Common Terminology Criteria for Adverse Events classification version 5.0 (CTCAE). Pathological response was assessed according to the peritoneal regression grading score (PRGS) and any reduction of the PRGS was considered a response to treatment. Each patient was recalled and followed up to death whenever possible. The Ethical Committee of the Foundation Policlinico A. Gemelli approved the study (ID: 2541; Prot. 16328/19), according to the principles of the Declaration of Helsinki. All patients provided written informed consent.

### PIPAC procedure

A PM interdisciplinary tumor board gave the indication for PIPAC on an individual basis considering several factors: ECOG PS, past chemotherapy lines and responses, previous surgery, clinical evaluation of abdominal accessibility, and disease extension on CT scan. Exclusion criteria were ECOG PS higher than 2, bowel obstruction, limited accessibility to the abdominal cavity on clinical evaluation, presence of other distant metastases, severe renal, hepatic or bone marrow impairment. The PIPAC procedure was performed according to the standard technique [21, 22]. An exploratory laparoscopy with peritoneal disease assessment with PCI was carried out. If present, ascitic fluid was drained and at least four peritoneal biopsies were taken for pathological response assessment. A nebulizer (Capnopen-MIP, Reger Medizintechnik, Rottweil, Germany) connected to a high-pressure injector (Injektron 82M, MedTron, Saarbrücken) creates a pressurized aerosol containing doxorubicin 1.5 mg/m^2^ body surface area in 50 mL of NaCl 0.9%, with cisplatin 7.5 mg/m^2^ in 150 mL NaCl 0.9% or oxaliplatin 92 mg/m^2^ body surface in 200 mL NaCl 0.9%. Since the beginning of 2020, cisplatin-doxorubicin dosages were updated to 10.5–2.1 mg/m^2^ on the basis of the dedicated dose-escalation study [23]. The injector flow is set to 6 mL/s with a maximum upstream pressure of 200 psi and a 12 mmHg capnoperitoneum. The injector flow is set to 30 mL/min with a maximum upstream pressure of 200 psi and an intraabdominal pressure of 12 mmHg. The injection is remote-controlled, and it is monitored by a laparoscopic camera held in place by a self-retaining retractor. The capnoperitoneum is then maintained for 30 min at 37 °C. After aerosol evacuation via a closed aerosol waste system, the trocars are removed. The fascia and skin were closed with absorbable sutures. Patients were discharged on the first or second postoperative day. A PIPAC course globally consists of three cycles, scheduled every 6–8 weeks.

### CT-derived body composition parameters

A specific image analysis software (SliceOmatic v5.0, Tomovision, Montreal, Canada) was used to examine CT images, by an operator trained in musculoskeletal anatomy, to define different tissues, according to the following Hounsfield Unit (HU) thresholds: −29 to +150 for muscle, −190 to −30 for intermuscular adipose tissue (IMAT), −150 to −50 for visceral adipose tissue (VAT), and −190 to −30 for subcutaneous adipose tissue (SAT). Skeletal muscle area (SMA) was analyzed on a single axial slice at the third vertebral level aiming to include following muscular groups: psoas, erector spinae, quadratus lumborum, transversus abdominis, external and internal obliques, and rectus abdominis. Muscle attenuation (MA) was obtained by the mean HU of SMA. Tissue boundaries were manually corrected as needed. Normalizing the previously measured parameters by height squared, skeletal muscle index (SMI), visceral adipose tissue index (VATI), and subcutaneous adipose tissue (SATI) were obtained, while the total fat area (TFA) was calculated adding all the fat tissues. According to previously published studies on PM patients [24, 25], 52.4 cm^2^/m^2^ for men and 38.5 cm^2^/m^2^ for women were used as cut-off values to define low-SMI patients.

### Statistical analysis

The objective of the study was to describe the immunonutritional status of patients undergoing PIPAC and to assess its relation with procedure-related, oncological and survival outcomes. In particular, we explored the immunonutritional variables related to the following endpoints: receiving multiple PIPAC cycles, PIPAC-related adverse events, pathological response on PM biopsies, and overall survival (OS). Kolmogorov–Smirnov test was used to assess normal distribution. Continuous variables were expressed as median (25 and 75th percentiles), categorical ones as number (percentage). Wilcoxon rank-sum test was used to assess differences between two groups; Chi-square or Fisher Exact test were appropriately used for categorical variables. ROC curves were used to find the cut-off of the parameters statistically significant at univariate analysis, reporting area under the curve (AUC), and cut off, were necessary. OS was calculated using Kaplan–Meier curves and differences between them were assessed through the log-rank test. All significant parameters at univariate analysis (p<0.05) were used to construct a Cox proportional regression analysis. Statistical analysis was performed using STATA^®^ Software (Version 14.0, Stata Corporation; College Station, TX, USA).

## Results

The baseline characteristics of the 51 patients are summarized in Table 1. The cohort study was composed of 26 males (51%) and 25 females (49%), with a median age of 63 years (54–71), and a median BMI of 20.9 kg/m^2^ (18.6–24.6). Fourty one patients (80.4%) were malnourished according to GLIM criteria. Five patients (10%) were in the third class of the ASA score, and 6 (11%) were in the second ECOG PS class. Primary tumors were gastric (39%), colorectal (33%), and hepato–pancreatic–biliary (HPB) (24%), with a 43% rate of synchronous PM. Almost all patients had already undergone one line of systemic chemotherapy, and 31 (60%) underwent two or more lines of systemic chemotherapy. PIPAC-related data are presented in Table 2. The access to the abdominal cavity and the first PIPAC cycle was feasible in all cases. Thirty (58.8%) patients repeated PIPAC procedure, and the median hospital stay was two days (1–3) without any readmission.

**Table 1:** Baseline characteristics of the study sample.

	n (%) or median (IQR)
Female	25 (49)
Age, years	63 (54–71)
Weight, kg	59 (52–71)
Height, cm	168 (163–173)
BMI, kg/m^2^	20.9 (18.6–24.6)
Malnourished according to GLIM	41 (80.4)
ASA score	
1	7 (14)
2	39 (76)
3	5 (10)
ECOG PS	
0	11 (22)
1	34 (67)
2	6 (11)
Primary neoplasm	
Colorectal	19 (37)
Gastric	20 (39)
HPB	12 (24)
Synchronous	22 (43)
Metachronous	29 (57)
Previous systemic chemotherapy	
None	1 (2)
1 line	50 (98)
≥2 lines	31 (60)

ASA, American Society of Anesthesiology; BMI, body mass index; ECOG PS, Eastern Cooperative Oncology Group performance status scale; GLIM, global leadership initiative on malnutrition; HPB, hepato-pancreatic-biliary cancer; IQR, interquartile range; PIPAC, pressurized intraperitoneal aerosol chemotherapy.

**Table 2:** Operative and postoperative PIPAC-related data.

	n (%) or median (IQR)
Total number of PIPAC	102
Only I PIPAC cycle	21 (41)
Multiple PIPAC cycles	30 (58)
Laparoscopic entry failures	0 (0)
PCI	22 (12–30)
Ascites, mL	500 (28–1.350)
Cisplatin–doxorubicin 7.5–1.5, mg/mq	28 (55)^a^
Oxaliplatin 92, mg/mq	20 (39)
Operative time, min	98 (74–131)
Intraoperative complications	1
Hospital stay, days	2 (1–3)
Readmission rate	0
Adverse events (CTCAE v. 5.0)	
Grades 1–2	17 (17)
Grade 3	1 (1)^b^
Grade≥4	0 (0)
Pathological response	28 (55)

IQR, interquartile range; PIPAC, pressurized intraperitoneal aerosol chemotherapy; CTCAE, common terminology criteria for adverse events. ^a^One patient underwent cisplatin 7.5 mg/mq only due to previous adverse reaction to doxorubicin; six patients received cisplatin-doxorubicin 10.5–2.1 mg/mq after dosage update in 2020. ^b^Skin effusion and abdominal pain due to trocar-site chemotherapy infiltration.

### Receiving multiple PIPAC cycles

Data regarding patients receiving multiple PIPAC cycles are shown in Table 3. In particular, 30 patients (58.8%) did not complete the third PIPAC cycle; the main reasons were disease progression (50.0%), no access to abdominal cavity (23.5%), patient’s refusal (14.5%), patients waiting for the next cycle (6.0%), complete pathological response (3.0%), others (3.0%). Median SMI was 42.3 cm^2^/m^2^ (37.6–49.7), with an incidence of low-SMI rate of 72.6%. No differences between patients who received one or more PIPAC cycles about body composition parameters were found. ALB, LYM, and PNI were lower in patients who received only one PIPAC, while NEU and NLR were higher. Cut offs were as follow: 27.5 for ALB, 3.55 for NEU, 0.90 for LYM, 36.5 for PNI, and 4.8 for NLR. AUC was higher for PNI and ALB with the value of 0.907 (p: 0.0001) and 0.911 (p: 0.0001), respectively.

**Table 3:** Multiple PIPAC procedures data.

	Total (51 patients)	1 PIPAC (21 patients)	≥2 PIPAC (30 patients)	p-Value	AUC	Cutoff	Sens	Spec
SMA, cm^2^	120.1 (102.5–136.3)	121.4 (96.3–133.5)	119.4 (106.1–141.2)	0.76				
SMI, cm^2^/m^2^	42.3 (37.6–49.7)	42.8 (35.2–51.2)	41.6 (38.1–49.4)	0.93				
Low-SMI rate	37 (72.6%)	14 (66.7%)	23 (76.7%)	0.52				
MA, HU	41.3 (37.5–47.8)	42.6 (35.4–48.1)	40.6 (37.5–47.1)	0.84				
VAT, cm^2^	36.6 (19.7–75.8)	33.0 (16.4–75.8)	39.5 (19.7–83.7)	0.64				
VATI, cm^2^/m^2^	13.1 (6.5–28.9)	10.2 (6.2–32.3)	13.2 (6.5–28.9)	0.76				
SAT, cm^2^	92.3 (60.3–148.4)	90.9 (45.1–186.2)	94.3 (62.3–119.6)	0.83				
SATI, cm^2^/m^2^	32.9 (19.8–49.6)	34.5 (16.1–59.4)	32.6 (22.2–43.4)	0.90				
IMAT, cm^2^	6.4 (2.7–8.4)	6.7 (3.5–8.4)	5.6 (2.5–8.7)	0.79				
TFA, cm^2^	149.8 (98.5–257.8)	160.5 (70.1–298.9)	140.1 (99.9–230.6)	0.85				
Malnutrition according to GLIM	41 (80.4%)	17 (80.9%)	24 (80.0%)	0.93				
Creatinine, mg/dL	0.85 (0.73–1.15)	0.86 (0.78–1.53)	0.83 (0.69–1.11)	0.35				
Albumin, g/L	29 (22–36)	22 (20–23)	33.5 (29–37)	**<0.0001**	0.907	27.5	87	90
Neutrophils, 10^3^ cells/mm^3^	4.26 (2.60–5.32)	4.51 (4.10–7.50)	3.45 (2.43–5.00)	**0.03**	0.679	3.55	53	86
Lymphocytes, 10^3^ cells/mm^3^	1.04 (0.76–1.48)	0.83 (0.67–1.29)	1.23 (0.86–1.57)	**0.02**	0.687	0.90	73	62
Platelets, 10^3^ cells/mm^3^	199 (135–284)	213 (123–311)	197 (141–276)	0.77				
PNI	34.9 (26.2–42.1)	25.9 (24.3–28.5)	40.7 (35.5–44.6)	**<0.0001**	0.911	36.5	97	86
NLR	4.6 (2.1–6.7)	6.2 (5.1–7.4)	2.4 (1.8–4.7)	**0.001**	0.771	4.8	77	81
PLR	179.2 (128.8–276.5)	252.1 (138.2–428.8)	175.7 (108.7–275.8)	0.12				

Data in bold indicate a statistically significant association. AUC, area under the ROC curve; GLIM, global leadership initiative on malnutrition; IMAT, intermuscular adipose tissue; MA, muscle attenuation; NLR, neutrophil-to-lymphocyte ratio; PIPAC, pressurized intraperitoneal aerosol chemotherapy; PLR, platelet-to-lymphocyte ratio; PNI, prognostic nutritional index; SAT, subcutaneous adipose tissue; SATI, subcutaneous adipose tissue index; Sens, sensitivity; SMA, skeletal muscle area; SMI, skeletal muscle index; Spec, specificity; TFA, total fat area; VAT, visceral adipose tissue; VATI, visceral adipose tissue index.

### PIPAC-related adverse events

Of 102 total PIPAC procedures performed, 18 (17.6%) adverse events were developed, of which only 1 (0.9%) was Grade 3 according to CTCAE. The only severe AE consisted of diffuse abdominal cutaneous and subcutaneous inflammation due to the infiltration of oxaliplatin from the trocar sites at the second PIPAC cycle. There were no grade four and five adverse events. Due to the very limited number of severe AE, no further analysis was performed on this issue.

### Pathological response

A pathological response according to the PRGS was documented in 28 out of 30 patients receiving more than one PIPAC and available for evaluation, which accounts for 55% of the overall cohort. Table 4 reports data correlated to pathological response. No differences between responders and non-responders according to PRGS were found in terms of blood tests. MA was higher in responding half of patients, while VAT, VATI, SAT, SATI, and TFA were lower in the same population. Cut offs were as follow: 39.5 for MA, 35.4 for VAT, 13.1 for VATI, 89 for SAT, 32.1 for SATI, and 149.8 for TFA. The highest AUC value was for SAT (0.739; p: 0.005).

**Table 4:** Laboratory and body composition data correlated to pathological response.

	No pathological response (23 patients)	Pathological response (28 patients)	p-Value	AUC	Cut off	Sens	Spec
SMA, cm^2^	119.3 (104.2–144.8)	121.3 (102.3–130.6)	0.62				
SMI, cm^2^/m^2^	42.4 (38.0–51.3)	42.0 (37.6–44.6)	0.73				
Low-SMI rate	15 (65.2%)	22 (78.6%)	0.35				
MA, HU	38.2 (33.7–43.7)	43.6 (40.4–48.5)	**0.02**	0.693	39.5	84	62
VAT, cm^2^	62.1 (34.8–86.9)	25.8 (15.5–57.7)	**0.02**	0.691	35.4	76	61
VATI, cm^2^/m^2^	22.4 (13.2–28.9)	9.5 (5.7–21.0)	**0.03**	0.688	13.1	75	77
SAT, cm^2^	118.3 (90.9–168.6)	74.6 (50.7–102.7)	**0.005**	0.739	89.0	71	82
SATI, cm^2^/m^2^	41.5 (32.6–59.4)	27.3 (17.4–38.6)	**0.007**	0.731	32.1	71	77
IMAT, cm^2^	7.1 (2.7–10.8)	5.4 (2.8–7.7)	0.24				
TFA, cm^2^	194.9 (124.2–290.7)	103.9 (77.6–175.8)	**0.01**	0.703	149.8	71	73
Malnutrition according to GLIM	19 (82.6%)	22 (78.6%)	0.72				
Haemoglobin, g/dL	11.8 (11.7–12.9)	12.8 (11.8–14.2)	0.39				
Creatinine, mg/dL	0.85 (0.76–1.07)	0.87 (0.69–1.51)	0.83				
Albumin, g/L	28 (20–34)	29 (22–38)	0.26				
Neutrophils, 10^3^ cells/mm^3^	4.36 (2.43–7.94)	4.10 (3.08–5.02)	0.62				
Lymphocytes, 10^3^ cells/mm^3^	1.11 (0.76–1.57)	1.03 (0.73–1.44)	0.51				
Platelets, 10^3^ cells/mm^3^	203 (129–282)	200 (152–289)	0.86				
PNI	34.9 (25.6–41.5)	35.2 (27.3–43.0)	0.55				
NLR	4.83 (1.92–8.37)	3.95 (2.94–5.45)	0.87				
PLR	238.3 (132.6–411.8)	186.4 (110.4–285.3)	0.64				

Data in bold indicate a statistically significant association. AUC, area under the ROC curve; GLIM, global leadership initiative on malnutrition; IMAT, intermuscular adipose tissue; MA, muscle attenuation; NLR, neutrophil-to-lymphocyte ratio; PIPAC, pressurized intraperitoneal aerosol chemotherapy; PLR, platelet-to-lymphocyte ratio; PNI, prognostic nutritional index; SAT, subcutaneous adipose tissue; SATI, subcutaneous adipose tissue index; Sens, sensitivity; SMA, skeletal muscle area; SMI, skeletal muscle index; Spec, specificity; TFA, total fat area; VAT, visceral adipose tissue; VATI, visceral adipose tissue index.

### Overall survival

**Figure 1:**
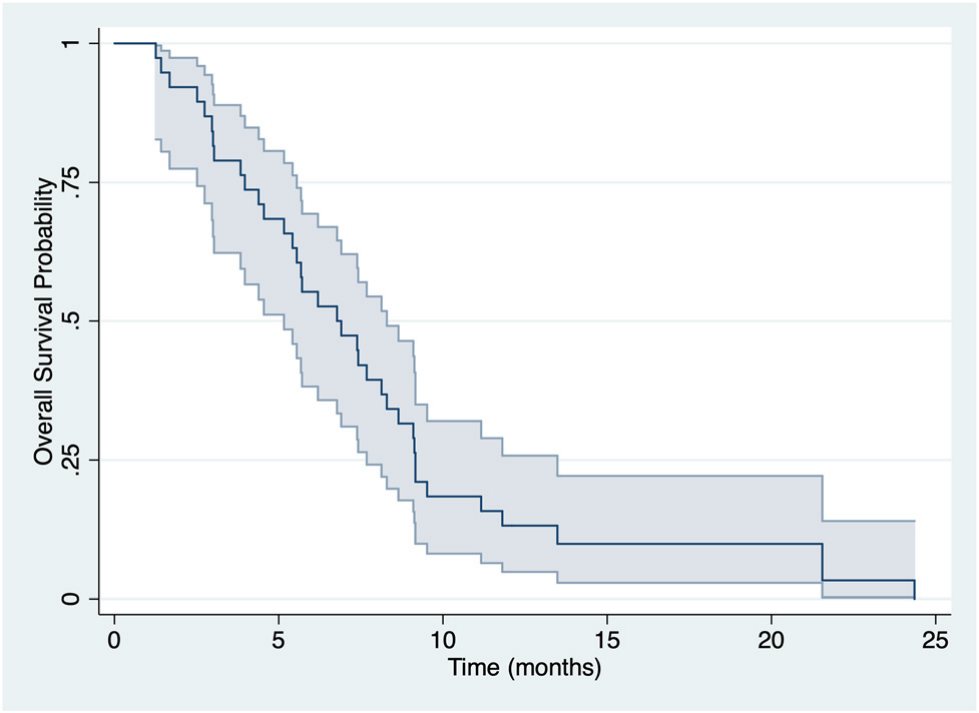
Overall survival analysis.

**Figure 2:**
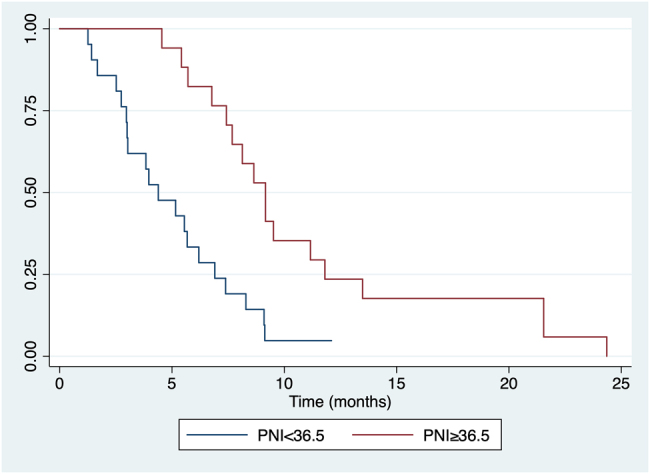
Univariate Kaplan–Meier analysis for PNI.

Within the median follow-up period of 36.0 months (range: 27.6–44.4), 38 (74.5%) patients died, with a median OS of 8.33 months (95% CI 5.90–9.47) (Figure 1). Table 5 reported univariate Kaplan–Meier analysis for all the tested variables and the Cox regression multivariate analysis performed. For PNI analysis the same cut off found in Table 3 was used. In particular, ascites [HR 2.50 (95% CI 1.17–5.30); p: 0.01], dysphagia [HR 2.83 (95% CI 1.11–7.19); p: 0.02], and PNI less than 36.5 [HR 3.43 (95% CI 1.65–7.15); p: 0.0005] resulted associated with a poor OS (Figure 2). At the Cox regression model, a low PNI [HR 2.41 (95% CI 1.08–5.46); p: 0.034] remained the only independent factor for OS.

**Table 5:** Univariate and multivariate analysis for overall survival.

	Univariate		Multivariate	
	HR (95% CI)	p-Value	HR (95% CI)	p-Value
Age≥65	1.49 (0.73–3.04)	0.26		
Sex	1.68 (0.86–3.28)	0.11		
BMI>18.5	0.83 (0.37–1.85)	0.64		
Malnutrition according to GLIM	1.08 (0.48–2.41)	0.85		
ECOG≥2	0.81 (0.25–2.66)	0.73		
ASA≥3	0.76 (0.23–2.49)	0.64		
Ascites	2.50 (1.17–5.30)	**0.01**	2.18 (0.91–5.24)	0.08
Dysphagia	2.83 (1.11–7.19)	**0.02**	3.17 (0.94–8.98)	0.07
Nausea	1.29 (0.60–2.78)	0.49		
CHT cycles≥12	0.65 (0.33–1.25)	0.18		
PNI<36.5	3.43 (1.65–7.15)	**0.0005**	2.41 (1.08–5.46)	**0.034**
Low-SMI rate	1.15 (0.56–2.39)	0.69		
MA	1.21 (0.61–2.43)	0.57		
PRGS	0.69 (0.36–1.35)	0.29		

Data in bold indicate a statistically significant association. ASA, American Society of Anesthesiology; BMI, body mass index; CHT, chemotherapy; ECOG PS, Eastern Cooperative Oncology Group performance status scale; GLIM, global leadership initiative on malnutrition; MA, muscle attenuation; PIPAC, pressurized intraperitoneal aerosol chemotherapy; PNI, prognostic nutritional index; SMI, skeletal muscle index.

## Discussion

To our best knowledge, this is the first study evaluating the impact of immunonutritional status and CT-derived body composition analysis on patients undergoing PIPAC for gastrointestinal PM. In fact, the only one study recently published, evaluating body composition parameters in PM patients, used bioelectrial impedance analysis rather than CT scan derived analysis [26].

PIPAC is a novel method of intraperitoneal chemotherapy administration with promising results for peritoneal surface malignancies, but, despite its safety and efficacy data are available, a precise indication is still under debate [27]. One of the most appealing features of PIPAC is the possibility to undergo multiple cycles and thus progressively hit a higher quantity of tumor cells. Since only half of patients are able to complete the programmed treatment cycle [9, 28], there is a need to find new objective selection criteria to decrease the number of patients undergoing only one PIPAC.

In our study, 58% of patients received multiple PIPAC cycles, in line with literature data [5, 29]. Among the pre-treatment immunonutritional parameters assessed, ALB, NEU, and LYM were significantly associated with the capability for PM patients to tolerate more than one PIPAC. Moreover, for their derived indexes PNI and NLR, cut offs of 36.5 and 4.8 were respectively calculated, but, due to the small sample size, they need further validation on a larger and more selected population. PNI is an unexpensive and easy to calculate parameter, which reflects the nutrition and inflammatory status of patients [30], whose role as a prognostic index for OS in many oncological settings has been widely recognised [31], [32], [33]. In our study, a PNI under the identified cut off value (36.5) resulted the only independently correlated factor with a worse OS (HR 2.41), and similar results were reported in a recent large retrospective study conducted on PM patients [34]. Starting from these findings, an immunonutritional evaluation should be routinely performed before programming a PIPAC treatment, and patients with an impaired status referred to a clinical nutrition unit.

A compromised nutritional status in PM patients, mostly depending on both mechanical and metabolic factors, can determine a body composition impairment [35]. In the last decade there has been an increasing interest in body composition analysis in cancer patients, since a quantitative and qualitative impairment of skeletal muscle mass determines a limited patient capacity to cope with surgical and antiblastic treatments [36, 37]. In fact, in almost all oncological settings, a low skeletal muscle mass, derived from SMI, is considered an independent prognostic factor for increased postoperative morbidity, chemotherapy toxicity, and worse OS [38, 39]. As previously reported, patients’ selection for PIPAC depends on several factors, including immunonutritional status, and in this study was defined after an interdisciplinary tumor board. The absolute contraindications to PIPAC were known hypersensitivity reaction to antiblastic agents, advanced metastatic disease with clinical deterioration and clinical or radiological evidence of gastrointestinal occlusion. To date, in literature there are no well-defined nutritional criteria or symptoms that preclude PIPAC procedure, so this field should be further investigated in next studies. In the complexity of this scenario, in which PIPAC improves survivals of PM patients, enhances quality of life, and relieves symptoms related to PM [40], the ideal patient for PIPAC is everyone who completes a three-cycle course in order to maximize the effects of this therapy. Consequently, an adequate nutritional support initiated at an earlier stage could improve clinical outcomes and treatment compliance, particularly in malnourished patients with metastatic disease.

While in previously published studies in gastrointestinal PM patients, the incidence of low SMI was 40–55% [24, 25, 40–42], in our study there was a higher prevalence of low SMI patient, reaching 73%. However, no SMI differences were found among the analyzed outcomes, while MA, describing the quality of muscle tissue, was correlated to pathological response after PIPAC, predicting a reduction in PRGS score on subsequent peritoneal biopsies. Also other studies on advanced ovarian cancer correlated MA, but not SMI, with oncological and survival outcomes [43, 44], introducing the possibility that in PM patients muscle quality might be considered a better predictor than muscle quantity. The CT scan analysis of fat tissues in PM patients showed that lower VAT, SAT, and TFA values were correlated to a better pathological response. Our hypothesis is that a higher quantity of fat mass, above all VAT, can act as a physical barrier for chemotherapeutic agents nebulized with PIPAC, reducing in the short term the pathological response.

This study is affected by several limitations: i) the sample size needs to be increased to confirm our findings; ii) since changes in body composition during chemotherapy administration are frequently reported [45], [46], [47], further longitudinal analysis is planned to investigate the impact of repeated PIPAC cycles; iii) GI primary tumors may differently condition nutritional status of PM patients.

In conclusion, the European Society of Clinical Nutrition Guidelines on cancer patients [48, 49] suggest a nutritional evaluation at the beginning of any oncological pathway to early identify patients at risk of malnutrition and those already malnourished, in which a nutritional intervention could reduce the risk of therapies discontinuation [50]. Since PIPAC procedure is administrated in very advanced oncological patients, an accurate nutritional evaluation is even more advisable. Further studies on a larger population of gastrointestinal PM patients receiving PIPAC are needed both to define the optimal selection criteria and to identify the best nutritional support to sustain them during the procedures.
